# Biomechanical Gait Variable Estimation Using Wearable Sensors after Unilateral Total Knee Arthroplasty

**DOI:** 10.3390/s18051577

**Published:** 2018-05-15

**Authors:** Ik-Hyun Youn, Jong-Hoon Youn, Joseph A. Zeni, Brian A. Knarr

**Affiliations:** 1 Division of Navigation & Information Systems, Mokpo National Maritime University, Mokpo 58628, Korea; 2 Department of Computer Science, University of Nebraska Omaha, Omaha, NE 68182, USA; jyoun@unomaha.edu; 3 Department of Physical Therapy, University of Delaware, Newark, DE 19716, USA; jzenijr@udel.edu; 4 Department of Biomechanics, University of Nebraska Omaha, Omaha, NE 68182, USA; bknarr@unomaha.edu

**Keywords:** biomechanical gait variable estimation, inertial gait variable, total knee arthroplasty, wearable sensors

## Abstract

Total knee arthroplasty is a common surgical treatment for end-stage osteoarthritis of the knee. The majority of existing studies that have explored the relationship between recovery and gait biomechanics have been conducted in laboratory settings. However, seamless gait parameter monitoring in real-world conditions may provide a better understanding of recovery post-surgery. The purpose of this study was to estimate kinematic and kinetic gait variables using two ankle-worn wearable sensors in individuals after unilateral total knee arthroplasty. Eighteen subjects at least six months post-unilateral total knee arthroplasty participated in this study. Four biomechanical gait variables were measured using an instrumented split-belt treadmill and motion capture systems. Concurrently, eleven inertial gait variables were extracted from two ankle-worn accelerometers. Subsets of the inertial gait variables for each biomechanical gait variable estimation were statistically selected. Then, hierarchical regressions were created to determine the directional contributions of the inertial gait variables for biomechanical gait variable estimations. Selected inertial gait variables significantly predicted trial-averaged biomechanical gait variables. Moreover, strong directionally-aligned relationships were observed. Wearable-based gait monitoring of multiple and sequential kinetic gait variables in daily life could provide a more accurate understanding of the relationships between movement patterns and recovery from total knee arthroplasty.

## 1. Introduction

Osteoarthritis (OA) of the knee is a common disease that impacts functional mobility and quality of life for many individuals [1,2]. Total Knee Arthroplasty (TKA) is the most common surgical treatment for end-stage OA. By replacing the impaired knee joint with implants, knee joint function and quality of life can be improved [2]. Since a primary goal of TKA is to regain ambulatory function, assessment of recovery after unilateral TKA using biomechanical gait variables can be useful clinical indicators. Monitoring the improvement of functional performance after TKA usually includes examining flexion and adduction knee moments and directional ground reaction forces [3]. Typically, studies on gait patterns before and after TKA use overground force plates and optical motion capture systems to collect biomechanical data [4]. While these instruments produce valid biomechanical data, they are constrained to laboratory settings and may not reflect real-world mobility patterns.

Wearable sensor-based continuous gait analysis is a promising alternative that can address the limitations of laboratory-based biomechanical evaluations [5]. Wearable devices are getting smaller and smarter, and wearables are easy to use and do not interfere with the natural behavior of the subject. Thus, wearable sensor-based gait analysis is one of the most promising methods for quantifying gait patterns in real-world conditions. Acceleration data from wearable sensors can also provide kinetic attributes outside of the laboratory setting. Monitoring multiple and sequential kinetic parameters using wearables in real-world conditions may lead to a more accurate understanding of the relationship between movement patterns and recovery from TKA.

Given the potential of wearable sensor-based gait analysis and the importance of gait outcomes post-TKA, the purpose of this study was to estimate biomechanical gait metrics using two wearable sensors in individuals after unilateral TKA. Biomechanical variables related to initial loading behavior (the initial 10% of the gait cycle) were selected since they are correlated with OA progression [6]. Concurrently, multiple inertial gait variables including a temporal parameter and kinetic parameters were extracted using wearable sensors. Then, the inertial gait variables were used to develop statistical prediction models of the selected biomechanical gait variables, such as moments and ground reaction forces, in the TKA population. Two main contributions of the present work are: (1) a generic method for linear inertial gait variable extraction; and (2) statistical models for estimation of important biomechanical gait variables.

## 2. Materials and Methods

**Figure 1:**
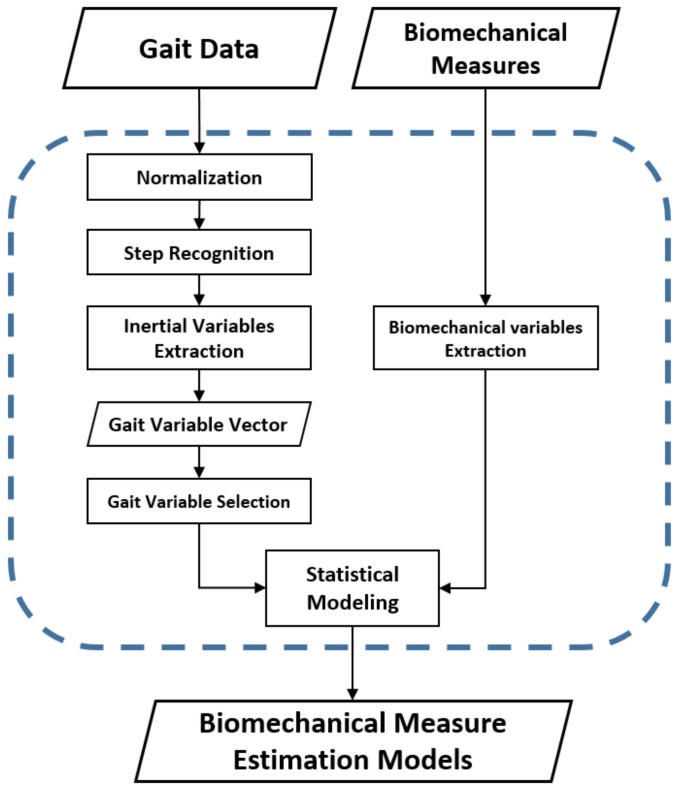
Framework for developing proposed biomechanical measure estimation models.

The framework in Figure 1 outlines the entire method of this study from data collection to estimation model development. Due to the importance of the initial loading characteristics during the initiative gait cycle of the TKA population [6], gait variables related to initial loading behavior were carefully considered when both biomechanical and inertial gait variables were selected. Feature selection was performed to obtain statistically meaningful inertial gait variable subsets, and hierarchical linear regressions were created to determine the directional contributions of inertial gait variables to the key biomechanical gait variables of the TKA population.

### 2.1. Data Description

**Figure 2:**
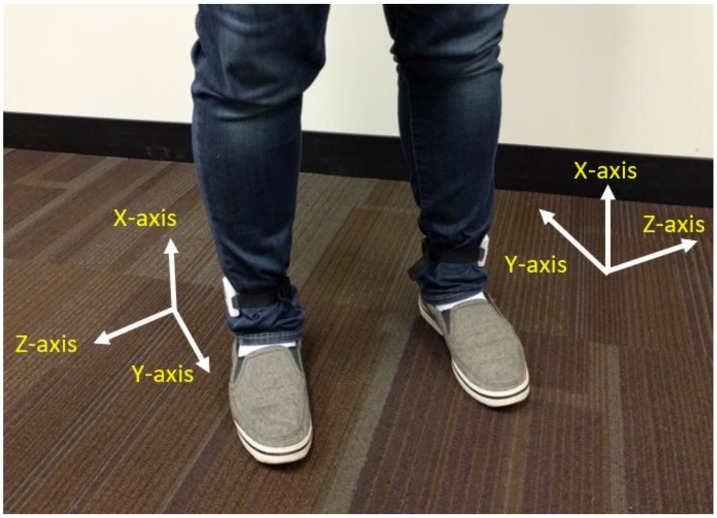
Wearable sensor orientation on both legs.

Data were acquired in the Neuromuscular Biomechanics Laboratory at the University of Delaware. An instrumented split-belt treadmill (Bertec Corp, Columbus, OH, USA) and an 8-camera motion capture system (Motion Analysis Corp, Santa Rosa, CA, USA) were used to collect kinetic and kinematic gait variables. Concurrently, two accelerometers (Noraxon USA, Scottsdale, AZ, USA) were attached above the lateral malleoli using elastic bands to collect three-dimensional acceleration data. The sampling frequency of acceleration data was 200 Hz. Figure 2 demonstrates the orientation of both wearable sensors. For both legs, the X-axis of the sensors pointed up to the shank, but the Y-axis and Z-axis orientations pointed in different directions on the left and right legs. The biomechanical data from the force place, and the cameras and inertial data from the wearables, were synched via hardware trigger. Biomechanical gait variables and inertial gait variables were computed using Visual 3D (C-Motion, Bethesda, MD, USA) and custom software developed in the MATLAB 9.0 (Mathworks, Natick, MA, USA) environment, respectively.

### 2.2. Participants and Protocol

Eighteen subjects (1.71 ± 0.08 m, 87.1 ± 17.5 kg, 66.5 ± 7.7 years) after unilateral TKA participated in this study. The study was approved by the Institutional Review Board at the University of Delaware. Each participant signed an informed consent before commencing protocols. Participants at least six months post-unilateral TKA were recruited (Table 1). Each subject conducted a 6-m walk test to determine a self-selected walking speed. Then, each participant was instructed to walk at a self-selected, comfortable walking speed on the instrumented split-belt treadmill for one minute. Participants walked 1.11 (±0.19) m/s during the one-minute walk test. 

### 2.3. Normalization

Kinetic and kinematic gait variables are often affected by the height and weight of the subjects. Acceleration from the lower limb may also be affected by these same factors. To minimize the confounding effects of patient anthropometric differences, we normalized both biomechanical and inertial gait variables by relevant factors. In prior research, Moisio et al. found that normalization methods were highly effective in reducing individual differences [7]. To normalize personal differences, kinetic and kinematic gait signals were divided by weight, and acceleration data were divided by height.

### 2.4. Biomechanical Gait Variable Extraction

In this session, we present information on the processes used to obtain the biomechanical gait variables of interest from laboratory instruments. Biomechanical gait variables included kinetic parameters such as knee moments and ground reaction forces. The process of feature extraction is detailed below. In this study, we focused on initial loading behavior-related inertial gait variables as important recovery indicators for the population. Loading characteristics during gait are important as they are correlated with OA progression [6]. To address the loading patterns, relevant kinetic biomechanical gait variables were measured including the maximum knee flexion moment (KFM), the maximum knee adduction moment (KAM), the first peak of vertical ground reaction force (vGRF) [8,9]. Additionally, the maximum anterior ground reaction force (aGRF) was included since the parameter has been used for various knee moment studies [10]. Although aGRF has less association with initial loading, the prediction of aGRF was expected to provide us information about the validity of our approach. 

**Figure 3:**
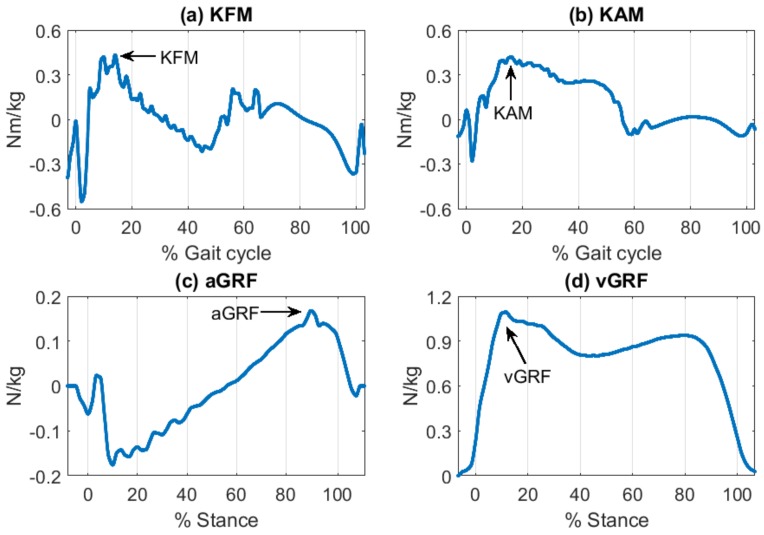
Kinetic biomechanical gait variables: weight-normalized. (**a**) Knee flexion moment (KFM); (**b**) knee adduction moment (KAM); (**c**) anterior/posterior ground reaction forces (aGRF); (**d**) vertical ground reaction force (vGRF).

Each step was first recognized by the point where 20% of the maximum vertical ground reaction force occurred [11]. Then, intended patterns (i.e., maximum or first peak maximum) were recognized to obtain targeted biomechanical gait variables (Table 2 and Figure 3). Since both the average and symmetry of biomechanical gait variables are important indicators for unilateral TKA [12,13], the four biomechanical gait variables from each step were then summarized in terms of average and symmetry across the one-minute trial. On average, 103 (±27.4) steps were included from the trial. Symmetry was calculated for each stride, and data from all steps were averaged when calculating average parameters.

### 2.5. Inertial Gait Variable Extraction

**Figure 4:**
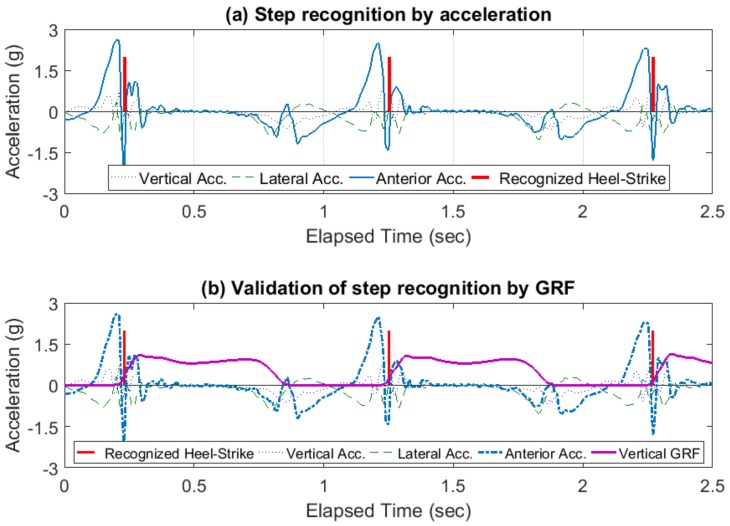
Step detection and validation with recognized heel-strikes. (**a**) Step detection using acceleration in the anterior/posterior direction; (**b**) validation of step recognition using the ground reaction force in the vertical direction. Note that the intervals between recognized heel-strikes indicate stride cycles.

Anterior directional acceleration was selected for accurate gait event recognition since the anterior dimensional motion of the lower limbs was dominant over the two other dimensions from an ankle-worn sensor perspective. Each heel-strike action generated a dramatic peak in the anterior directional acceleration; this peak was a clear indicator of the initial loading within a gait cycle (Figure 4a). The identified peaks were compared to the vertical ground reaction force data to validate the accuracy of acceleration-based gait event detection (Figure 4b). The described methodology was applied to each ankle sensor individually. Once individual step recognition was complete, the recognized peaks from the two sensors and raw acceleration data were merged together to obtain data on step cycles.

Eleven gait variables were extracted to estimate the magnitude, impulse, and angles of initial loading from 3D-acceleration data. Since the focus of this study was on the initial loading characteristics of TKA patients, the inertial motion of the lower limbs following heel-strike (HS) was analyzed. Characteristics from the initial 10% of the stance phase of the gait cycle, the initial 10% of the directional impulse of the gait cycle, and the maximum directional acceleration at HS were extracted. Additionally, whole step vector magnitude, ankle angle variation in the lateral and anterior directions, and step time were computed to explain the characteristics of the whole step cycle (Table 3).

The vectors of the basic gait variables from each step were summarized in terms of average and symmetry for each trial. The trial-averaged inertial gait variables were applied to estimate the biomechanical gait variables. Since bilateral gait symmetry has gained more attention, particularly in the unilateral TKA population [9,13], the basic gait variables were used to calculate symmetry. The Symmetry Index (SI) proposed by Robinson et al. was applied to assess the symmetry of inertial gait variables [14]. To apply the concept of SI for TKA patients, SI was defined in the study as the difference between the non-surgical limb from the surgical limb, rather than the difference between the left limb and the right limb.

### 2.6. Data Analysis

To quantify the relationships between all independent (i.e., eleven inertial gait variables) and dependent variables (i.e., four biomechanical gait variables), a Pearson Correlation analysis was conducted. For the statistical analysis, the eleven inertial gait variables were categorized by directional perspectives, i.e., lateral, anterior, vertical, and inclusive inertial gait variables (Table 4).

To avoid overfitting the estimation models, subsets of eleven inertial gait variables were carefully selected for each of the four biomechanical gait variables as a preprocessing method. Stepwise regression was applied to systematically select relevant inertial gait variables for the four biomechanical gait variables [15]. The automatic procedure of stepwise regression of feeding all useful inertial variables helped us to reduce the amount of mutual information (i.e., non-overlapping) among eleven independent variables- with smaller subset sizes. The stepwise regression criterion for variable inclusion was an increase in the adjusted R^2^ value. To improve the robustness of the model, k-fold cross-validation [16] was applied with k = 10. In k-fold cross-validation, 18 participants were randomly partitioned into 10 subfolders. A single subfolder was retained as the validation data for testing the model, and the remaining nine subfolders were used as training data. The cross-validation process was then repeated 10 times, with each subfolder used exactly once as the validation data. The procedure was intended to make estimation models robust enough to be for unseen TKA patients’ gait data and to improve the overall validity of model predictions.

To determine the directional contributions to biomechanical measures, hierarchical linear regressions were used [16]. Specifically, selected inertial variables in each directional category were added to the regression models in steps as discussed in [17]. This procedure provided information regarding which directional inertial variables had the most predictive power on the biomechanical variable estimation models. Separate regressions were conducted for each of the four biomechanical gait variables. Primary axis inertial variables were entered into the regressions at the first step. For example, KFM was knee moment in the anterior–posterior direction, so the anterior direction inertial variables from feature selection outcomes were added to the KFM estimation regression model in the first step. Then, the vertical and lateral inertial variables were entered in the second and third steps, respectively. For all hierarchical regressions, the inclusive inertial variables were entered at the last step. The order of hierarchical regression steps was determined in a cyclic way depending on directional aspects of target biomechanical variables. For example, to establish the KFM estimation model, anterior variables were entered first, then vertical and lateral inertial variables were additionally entered first, then vertical and lateral inertial variables were additionally entered, respectively. Inclusive variables were always entered last. The significance of each model and the significant change in R^2^ at each step were evaluated. The change in R^2^ provided increased predictive power through the addition of certain directional inertial variables at each regression step. 

## 3. Results

Overall, ten inertial variables were significantly correlated with KFM, aGRF, and vGRF (Table 5). Only the step time (ST) was not significantly correlated with any biomechanical variables. In particular, the vertical heel-strike impulse (IMP-V) was solely correlated with KAM. The selected inertial gait variable subsets for each of the four biomechanical gait variables are listed in Table 6 and Table 7.

For the trial-averaged biomechanical variable prediction models, no lateral inertial variables were selected for KFM and aGRF, and none of the vertical inertial variables were selected for KAM and vGRF (Table 6). ST was selected for all four biomechanical variable estimations, although ST was not significantly correlated with them in the Pearson Correlation analysis results. For the trial symmetry of biomechanical variable prediction models, the lateral and vertical magnitude variables (i.e., MAG-L, and MAG-V) were selected except the anterior magnitude variable (i.e., MAG-A) (Table 7). The inclusive variables were relatively less frequently selected for symmetry prediction models. The robustness and generalizability of the estimation models were improved by reducing the dimensionality of the inertial gait variables. 

The hierarchical linear regression results demonstrated a strong indication that the proposed wearable sensor-derived acceleration data could assist in quantifying biomechanical gait measures. In Table 8 and Table 9, the average and symmetry of the biomechanical variables were predicted using the selected inertial variables.

Each individual table contains the prediction results for 17 subjects. One subject (71-year-old male, BMI 31.6) was excluded from the analysis due to a distinctly abnormal gait pattern characterized by heel-strikes with overtly large vertical ground reaction forces. The subject was identified as an outlier based on the median absolute deviation measure. By removing this subject, the average and symmetry of the vGRF prediction showed a more reasonable prediction power. Regarding the average prediction results, all four biomechanical variables were significantly predicted using the selected subsets of inertial variables. Directional contributions were identified. For instance, aGRF was primarily related to the anterior axis, and the anterior inertial gait variables predicted most of the outcomes (i.e., 0.467 of 0.697 as adj. R^2^). Similar directional alignments were observed from KFM and KAM. Although vGRF was significantly predicted, there was no such directional alignment because none of the vertical inertial variables were selected. In the symmetry prediction outcomes, the selected inertial variables were significantly correlated with the symmetry of KFM. The effect of the uncommon walking subject was also trivial, so the exclusion of the subject did not change the results. Specifically, the symmetry of vGRF was substantially affected by the uncommon walking subject. The subject caused strongly biased gait variables and abnormally increased adj. R^2^ values of up to 0.919. By removing the subject, adj. R^2^ was adjusted by 0.547.

## 4. Discussion

The goal of this study was to estimate kinematic and kinetic gait metrics using two ankle-worn wearable sensors in individuals after unilateral TKA. Overall, we found that our novel method of extracting unique features from 3D accelerations was capable of predicting key biomechanical measure in a post-TKA population.

Compared to previous studies which focused on predicting knee loads post-TKA, our results demonstrated a greater predictive power. Rivière et al. focused on isolated clinical measures, such as limb alignment (R^2^ < 0.13) [3], and Vahtrick et al. investigated limb strength (R^2^ < 0.32) [4]. The results of this study indicate that wearable sensors can be used to predict key knee loading [1,2] variables important to recovery post-TKA with greater power than basic clinical measures. This may be due to the more direct nature of wearable accelerometry during gait, versus indirect measures of predisposition (limb alignment) or capacity (strength) that do not take into account an individual’s active movement and muscle coordination during the specific task of gait. 

The outcomes of the regression models indicated that inertial gait features significantly estimated all four biomechanical gait features. Interestingly, the temporal parameter of step time was not significantly correlated with any biomechanical variables of interest, whereas most of the inertial variables showed moderate to significant correlations with biomechanical variables. In particular, as the anterior direction motion of ankle-worn sensors was predominant over the other two directions, many inertial variables were significantly corrected with aGRF. 

The primary axes of biomechanical variables were related to selected inertial variables. However, it was difficult to explain the connection between response and predictor variables due to the complex nature of gait. Notably, lateral and vertical heel-strike magnitude and anterior stance phase angle variation were commonly selected for symmetry prediction models, and inclusive variables were considered to be less important predictor variables. Our results imply that wearable sensor-based data that explains overall step timing were not useful to estimate the symmetry of biomechanical variables. 

Knee flexion moment was primarily predicted by vertical inertial gait variables. It is likely that vertical inertial variables are related to limb impact during heel strike. The impact may be partially controlled by knee flexion, with an increase in knee flexion during weight acceptance serving to soften the impact but subsequently, increasing peak knee flexion moment. On the other hand, knee adduction moment was primarily predicted by lateral inertial gait variables. Gait modifications including increased step width, increased trunk sway, and toe-in gait have been shown to be effective for reducing the knee adduction moment in a healthy population. It is likely that individuals post-TKA may adapt similar strategies to reduce the knee adduction moment because of pain or functional compensations. It is reasonable to believe that such gait adaptations may be evidenced through lateral inertial gait variables, given the changes in side-to-side movement (i.e., swaying, wide steps).

## 5. Conclusions

The proposed models and biomechanical gait variable estimation results provided evidence that inertial measurements can be used to reasonably estimate conventional biomechanical metrics. Although cross-validation was applied, generalization to the TKA population could be limited due to the small sample size of the study. Future work will examine the relationship between additional kinematic and kinetic variables and inertial variables to characterize changes over time and to expand to additional populations and biomechanical metrics.

## Figures and Tables

**Table 1:** Participants’ characteristics.

Characteristics	Mean (Standard Deviation)
*n*	18
Female/male	9/9
Age (years)	66.5 (7.7)
Body Mass Index (BMI, kg/m^2^)	29.5 (4.9)
Height (cm)	171.4 (8.4)
Weight (kg)	87.1 (17.5)

**Table 2:** Biomechanical gait variable properties.

Purpose	Acronym	Description
Knee Flexion Moment	KFM	Maximum anterior knee moment
Knee Adduction Moment	KAM	Maximum lateral knee moment
Anterior Ground Reaction Force	aGRF	Maximum anterior direction GRF
Vertical Ground Reaction Force	vGRF	Maximum first vertical GRF

**Table 3:** Inertial gait variable properties.

Purpose	Acronym	Description	Method
Step magnitude	VM	Whole step vector magnitude	Residual acceleration during the step
Initial step magnitude	VM10	Step magnitude of initial 10% of the step time	Initial 10% of residual acceleration during the step
Directional magnitude of initial loading	MAG-L	Lateral heel-strike magnitude	Maximum lateral acceleration at HS
	MAG-V	Vertical heel-strike magnitude	Maximum vertical acceleration at HS
	MAG-A	Anterior heel-strike magnitude	Maximum anterior acceleration at HS
Directional impulse of initial loading	IMP-L	Lateral heel-strike impulse	SD of lateral acceleration during initial 10% of step
	IMP-V	Vertical heel-strike impulse	SD of vertical acceleration during initial 10% of step
	IMP-A	Anterior heel-strike impulse	SD of anterior acceleration during initial 10% of step
Directional ankle angle variation during stance phase	ANG-L	Ankle angle change to the gravity in the lateral direction during the stance phase	SD of sensor local angle change in lateral direction during stance phase
	ANG-A	Ankle angle change to the gravity in the anterior direction during the stance phase	SD of sensor local angle change in lateral direction during stance phase
Temporal parameter	ST	Step time	HS-to-HS time

HS is heel-strike; SD is standard deviation.

**Table 4:** Directional categories of inertial gait variables.

Directional Category	Description	Variable
Inclusive	Non-directional variables	VM, VM10, ST
Lateral	Lateral variables	MAG-L, IMP-L, ANG-L
Vertical	Vertical variables	MAG-V, IMP-V
Anterior	Anterior variables	MAG-A, IMP-A, ANG-A

**Table 5:** Pearson correlation coefficient between inertial and biomechanical variables.

	KFM	KAM	aGRF	vGRF
VM	0.63 **	-	0.74 **	0.62 **
VM10	0.60 **	-	0.79 **	0.67 **
MAG-L	-	-	0.51 *	-
MAG-V	-	-	0.52 *	-
MAG-A	0.73 **	-	0.74 **	0.55 **
IMP-L	0.59 **	-	0.74 **	0.54 *
IMP-V	-	−0.58 **	-	-
IMP-A	0.51 *	-	0.65 **	0.58 **
ANG-L	0.47 *	-	0.61 **	0.58 **
ANG-A	0.60 **	-	0.71 **	0.66 **
ST	-	-	-	-

** Correlation is significant at the 0.01 level; * correlation is significant at the 0.05 level; those with significance greater than 0.05 were removed.

**Table 6:** Feature selection results for average prediction.

Biomechanical Variable	Selected Inertial Gait Variables			
	Lateral	Vertical	Anterior	Inclusive
KFM	None	MAG-V	ANG-A	ST
KAM	IMP-L, MAG-L ANG-L	None	MAG-A	VM10 ST
aGRF	None	MAG-V IMP-V	MAG-A ANG-A	ST
vGRF	IMP-L, MAG-L ANG-L	None	IMP-A	VM10 ST

**Table 7:** Feature selection results for symmetry prediction.

Biomechanical Variable	Selected Inertial Gait Variables			
	Lateral	Vertical	Anterior	Inclusive
KFM	MAG-L IMP-L	MAG-V	IMP-A ANG-A	VM, VM10, ST
KAM	MAG-L	MAG-V IMP-V	IMP-A	None
aGRF	MAG-L	MAG-V	ANG-A	VM
vGRF	MAG-L	MAG-V	ANG-A	None

**Table 8:** Hierarchical linear regressions for averages of biomechanical gait variables.

**(a) Average Knee Flexion Moment (KFM)**					
**Step**	**All subjects**			**Excluding outlier**	
	**R**	**Adj. R^2^**	**∆ R^2^**	**Adj. R^2^**	**∆ R^2^**
Anterior	0.605	0.326	0.326	0.350	0.350
Vertical	0.740	0.486	0.160	0.442	0.092
Lateral	0.740	0.486	0.000	0.442	0.000
Inclusive	0.773	0.510	0.024	0.455	0.014
**(b) Average Knee Adduction Moment (KAM)**					
**Step**	**All subjects**			**Excluding outlier**	
	**R**	**Adj. R^2^**	**∆ R^2^**	**Adj. R^2^**	**∆ R^2^**
Lateral	0.664	0.321	0.321	0.319	0.319
Anterior	0.756	0.441	0.120	0.432	0.113
Vertical	0.756	0.441	0.000	0.432	0.000
Inclusive	0.867	0.615	0.175	0.614	0.182
**(c) Average Anterior Ground Reaction Force (aGRF)**					
**Step**	**All subjects**			**Excluding outlier**	
	**R**	**Adj. R^2^**	**∆ R^2^**	**Adj. R^2^**	**∆ R^2^**
Anterior	0.728	0.467	0.467	0.486	0.486
Vertical	0.846	0.629	0.162	0.614	0.128
Lateral	0.846	0.629	0.000	0.614	0.000
Inclusive	0.887	0.697	0.067	0.677	0.063
**(d) Average Vertical Ground Reaction Force (vGRF)**					
**Step**	**All subjects**			**Excluding outlier**	
	**R**	**Adj. R^2^**	**∆ R^2^**	**Adj. R^2^**	**∆ R^2^**
Vertical	0.000	0.000	0.000	0.000	0.000
Lateral	0.738	0.446	0.446	0.377	0.377
Anterior	0.748	0.425	−0.020	0.417	0.040
Inclusive	0.857	0.589	0.164	0.463	0.046

**Table 9:** Hierarchical linear regressions for symmetry of biomechanical gait variables.

**(a) Symmetry Knee Flexion Moment (KFM)**						
**Step**	**All subjects**			**Excluding outlier**	
	**R**	**Adj. R^2^**	**∆ R^2^**	**Adj. R^2^**	**∆ R^2^**
Anterior	0.680	0.391	0.391	0.373	0.373
Vertical	0.704	0.387	−0.004	0.431	0.058
Lateral	0.811	0.515	0.128	0.564	0.132
Inclusive	0.969	0.882	0.368	0.873	0.309
**(b) Symmetry Knee Adduction Moment (KAM)**						
**Step**	**All subjects**			**Excluding outlier**	
	**R**	**Adj. R^2^**	**∆ R^2^**	**Adj. R^2^**	**∆ R^2^**
Lateral	0.126	−0.046	−0.046	−0.055	−0.055
Anterior	0.173	−0.100	−0.054	−0.115	−0.060
Vertical	0.733	0.395	0.494	0.379	0.495
Inclusive	0.733	0.395	0.000	0.499	0.120
**(c) Symmetry Anterior Ground Reaction Force (aGRF)**						
**Step**	**All subjects**			**Excluding outlier**	
	**R**	**Adj. R^2^**	**∆ R^2^**	**Adj. R^2^**	**∆ R^2^**
Anterior	0.308	0.038	0.038	0.083	0.083
Vertical	0.488	0.136	0.098	0.014	−0.069
Lateral	0.501	0.090	−0.046	0.162	0.148
Inclusive	0.560	0.103	0.013	0.364	0.202
**(d) Symmetry Vertical Ground Reaction Force (vGRF)**						
**Step**	**All subjects**			**Excluding outlier**	
	**R**	**Adj. R^2^**	**∆ R^2^**	**Adj. R^2^**	**∆ R^2^**
Vertical	0.626	0.354	0.354	0.165	0.165
Lateral	0.765	0.529	0.175	0.222	0.057
Anterior	0.797	0.522	−0.007	0.473	0.251
Inclusive	0.971	0.919	0.397	0.547	0.074
